# Maternal cancer and congenital anomalies in children – a Danish nationwide cohort study

**DOI:** 10.1371/journal.pone.0173355

**Published:** 2017-03-06

**Authors:** Natalie C. Momen, Andreas Ernst, Linn Håkonsen Arendt, Jørn Olsen, Jiong Li, Mika Gissler, Finn Rasmussen, Cecilia Høst Ramlau-Hansen

**Affiliations:** 1 Department of Public Health, Section for Epidemiology, Aarhus University, Aarhus, Denmark; 2 Department of Clinical Epidemiology, Aarhus University Hospital, Aarhus, Denmark; 3 Department of Epidemiology, University of California Los Angeles, Los Angeles, CA, United States of America; 4 THL National Institute for Health and Welfare, Helsinki, Finland; 5 Karolinska Institute, Department of Neurobiology, Care Sciences and Society, Division of Family Medicine, Stockholm, Sweden; 6 Department of Health Sciences, Lund University, Lund, Sweden; Universidade de Sao Paulo, BRAZIL

## Abstract

Several studies on pregnancy-associated cancers have suggested an association with congenital anomalies in offspring. Previous studies have included maternal cancers diagnosed up to 2 years after pregnancy; however, long latency periods of some cancers mean that cancers diagnosed many years postpartum might have been present during pregnancy in a preclinical state. This paper considers the association between maternal cancers diagnosed from 2 years prior to pregnancy until the mother reaches 50 years of age, and congenital anomalies, as diagnosed at birth or within the first year of life. The current population-based study looks at associations of cancers in mothers with congenital anomalies in their children. Children were followed up from birth to diagnosis of a congenital anomaly, death, emigration or end of follow-up (whichever occurred first). A total of 56,016 children (2.6%) were considered exposed to a maternal cancer of any type; and they had a hazard ratio (HR) of 1.04 (95% confidence interval [CI]: 1.00, 1.09) compared with unexposed children. The greatest HR was seen among children whose mothers had been diagnosed with cancers before or during pregnancy (HR: 1.37, 95% CI: 1.07, 1.75). Similar results were seen when paternal cancers were used as a ‘negative control’. Statistically significant associations were seen for some specific congenital anomalies of organ systems (congenital anomalies of the musculoskeletal system [HR: 1.13, 95% CI: 1.02, 1.25]) and for some specific types of maternal cancer (leukaemia [HR: 1.31, 95% CI: 1.01, 1.61], The results of the main analyses suggest a small increase in risk of congenital anomalies in offspring of mothers diagnosed with cancer from 2 years before pregnancy, until the mother reaches 50 years of age; with the greatest increase seen for exposure in the pre-pregnancy and pregnancy period. These results may reflect shared causes for some cancers and some congenital anomalies. The similar results seen for paternal cancers indicate that the cause may be genetic or related to the families’ social and environmental conditions.

## Introduction

The prevalence of pregnancy-associated cancers is estimated at 1 in 1000 to 1 in 1500 of pregnancies [1–4]. The most common cancer forms affecting pregnant women are cervical cancers, breast cancers, melanomas, lymphomas, and leukaemias [1]. Generally, cancers diagnosed during or shortly after pregnancy, within one or two years after childbirth are considered to be pregnancy-associated cancers [5–8]; but, long periods can pass between an exposure initiating the growth of an abnormal cell, invasion of the cancer, symptoms becoming apparent, and a diagnosis and treatment being made. The minimum latency periods to clinical diagnoses for cancers in adults, were determined by the World Trade Center Health Program to be 0.4 years for lymphoproliferative and haematopoietic cancers and 4 years for solid tumours (excluding mesotheliomas [11 years] and thyroid cancers [2.5 years]) [9]. Additionally, a study using data on over 1.6 million cancers from the National Cancer Institute’s Surveillance, Epidemiology, and End Results (SEER) program concluded that most types of cancer progressed for 10 years or more before being detected [10]. It is also possible that some cancers have even longer induction and latency times than these. Therefore, cancers diagnosed postpartum may have been initiated, but remained undetected, during pregnancy, possibly affecting the developing fetus. Pregnancy-associated cancers, in this paper, are defined as all cancers diagnosed from 2 years prior to pregnancy and up to 50 years of age in the mothers.

The risk of congenital anomalies has been suggested to be greater in children born to mothers who were diagnosed with a pregnancy-associated cancer [11], including lymphomas [7] and breast cancer [12]; however, the results have not been consistent [5–7;13;14] and more studies are warranted. Three of these studies, looking at different types of cancers, were carried out using Danish data [5–7], considering cancers diagnosed up to two years after pregnancy. Further Danish studies have found that cancers (specifically, lymphomas and leukaemias) were more common among parents who had had a child born with a congenital anomaly [15] and that cancers and birth defects tend to cluster in families [16]. A few mechanisms through which pregnancy-associated cancers may increase the risk of congenital anomalies in the offspring have been suggested, however knowledge in this area is limited. Firstly, for cancers that are diagnosed during the gestational period, treatment regimens or psychological stress could impact risk of congenital anomalies [17–19]. Secondly, cancer development presents the body with metabolic [20;21], hormonal [21] and nutritional challenges [21–24], which could affect the growing conceptus. Further, it has been suggested that hyperthermia is more common among cancer patients [25], which may impact risk of congenital anomalies [6;26;27]. If the presence of a cancer coincides with pregnancy, some of these fetal challenges could have occurred even if a diagnosis was not received for several years after the pregnancy. Alternatively, there may be shared environmental or genetic factors influencing development of cancer in the mother and congenital anomalies in the offspring [15;16;21]. Even, if these exposures were present shortly before or during pregnancy, due to the induction and latency periods, the cancer may not be diagnosed for a number of years.

Since results from previous studies investigating the risk of congenital anomalies in children of mothers with pregnancy-associated cancer, with or without treatment during pregnancy, have been mixed [5–7;11;13;14], further research is needed on this topic, in particular using a larger cohort with longer follow-up time.

This study thus aims to investigate the association between maternal cancer and congenital anomalies in the offspring. In our study, exposure to maternal cancer is indicated by a diagnosis of cancer before the age of 50 years. Timing of the cancer diagnosis, in relation to the *in utero* period, will be considered in an attempt to separate the side effects of treatment from other fetal challenges related to the cancer itself. We hypothesised that there is a higher risk of congenital malformations among children whose mothers are diagnosed with cancer before or during pregnancy due to treatments and psychological stress; additionally, we hypothesised that there are increased risks among children whose mothers are diagnosed in the years soon after pregnancy, as these cancers would be most likely to have been present during the pregnancy.

## Methods

### Study participants and follow-up

All residents of Denmark are assigned a unique civil registration number, at birth or immigration, which can be used to accurately link data from the nationwide registers. We identified all children born from 1^st^ January 1977 to 31^st^ December 2008 in Denmark whose mother had not received a diagnosis of cancer prior to 2 years before conception. We obtained information on whether these children received diagnoses of congenital anomalies or their mothers received cancer diagnoses.

### Exposure

The main exposure of interest was any maternal cancers (International Classification of Diseases 7^th^ Revision [ICD-7] codes: 140–207 before 1994, ICD-10 codes: C00- C97 from 1994 to 2010) which were diagnosed from two years prior to the index child's birth, up to the mother reaching 50 years of age. As the risk of death is low up to 50 years of age, this cut off was chosen to reduce immortal observation time bias: if a mother died from a cause other than cancer before the cut-off point her child would be considered unexposed, despite the fact a diagnosis may have been made later, had she survived past her death date. Paternal cancer (diagnosed at the same time points) was considered as a 'negative control'. Maternal and paternal cancer diagnoses were extracted from the Danish Cancer Register. The registration and coding of cancers in Denmark have been described previously [28]. These data were linked to data in the Civil Person Register on the birth dates of the mother, to ascertain age at cancer diagnosis, and child, to limit to diagnoses in the exposure window.

Exposure was considered further by identifying the time point at which maternal (and paternal) cancer diagnoses were received: from 2 years prior to pregnancy, until the child's birth; within the 5 years postpartum; >5 years to 10 years postpartum; >10 years postpartum; or in multiple time points (diagnoses received in more than one of the previously described time points). However, exposure ascertainment extended only up until the mother reached 50 years of age. A sensitivity analysis used a cut-off of 45 years of age, to see whether this changed the results. As we expect numbers of cancers diagnosed from 2 years prior to pregnancy until the child’s birth to be small, we combined these groups for the main analyses, but we carried out additional analyses where these two time periods were considered separately (pre-pregnancy and pregnancy).

Additionally, type of cancer diagnosis according to the ICD classification grouping was considered: lip, oral cavity and pharynx (ICD-7: 140–148, ICD-10: C00-C14); digestive organs (ICD-7: 150–159, ICD-10: C15-C26); respiratory and intrathoracic organs (ICD-7: 160–165, ICD-10: C03-C39); bone and articular cartilage (ICD-7: 196, ICD-10: C40-C41); skin (ICD-7: 190–191, ICD-10: C43-C44); mesothelial and soft tissue (ICD-7: 197, ICD-10: C45-C49); breast (ICD-7: 170, ICD-10: C50); female genital organs (ICD-7: 171–176, ICD-10: C51-C58); urinary tract (ICD-7: 180–181, ICD-10: C60-C63); central nervous system (ICD-7: 192–193, ICD-10: C69-C72); endocrine (ICD-7: 194–195, ICD-10: C73-C75); other solid tumours (ICD-7: 198–199, ICD-10: C76-C80 and C97); leukaemias (ICD-7: 204, ICD-10: C91-C95); and lymphomas and other lymphoid and haemopoetic systems neoplasms (ICD-7: 196, ICD-10: C40-C41).

Due to some cancers clustering in families and some cancers being associated with particular congenital anomalies, we also carried out analyses with mother-child pairs removed if both had a diagnosis of cancer recorded in the Danish Cancer Register.

### Outcome

Information on diagnoses of congenital anomalies (ICD-8 codes 740–759 from 1977 to 1993, ICD-10 codes Q00-Q99 from 1994–2010) in the children was obtained from the Danish National Patient Register [29;30]. Offspring were followed up from birth to the first of the following events: diagnosis of a congenital anomaly, death, emigration or end of the data (December 31st 2010). The main analyses included diagnoses of congenital anomalies before the child reached 1 year of age, as these are expected to be more severe and the diagnoses more valid. In a sub-analysis congenital anomaly diagnoses made throughout the entire follow-up period were included, to assess whether this altered the results. Additionally, sub-analyses considered type of congenital anomaly: nervous system (ICD-8: 740–743, ICD-10: Q00-Q07); eye, ear or nose (ICD-8: 744–745, ICD-10: Q10-Q18); circulatory system (ICD-8: 746–746, ICD-10: Q20-Q28); respiratory system (ICD-8: 748, ICD-10: Q30-Q34); orofacial clefts (ICD-8: 749, ICD-10: Q35-Q37); digestive system (ICD-8: 750–751, ICD-10: Q38-Q45); genital organs (ICD-8: 752, ICD-10: Q50-Q56); urinary system (ICD-8: 753, ICD-10: Q60-Q64); musculoskeletal system (ICD-8: 754–756, ICD-10: Q65-Q79); other congenital anomalies and chromosomal abnormalities (ICD-8: 757–759, ICD-10: Q80-Q99).

### Covariates

We identified the following covariates in the Danish Medical Birth Register and the Integrated Database for Labour Market Research and included them in our models: maternal age at time of birth (15–26 years, 27–30 years, ≥31 years), paternal age at time of birth (15–26 years, 27–30 years, ≥31 years), birth year (continuous), offspring sex (male, female). Further models were run with the addition of socioeconomic variables recorded at time of the child’s birth: maternal highest education (lower secondary, higher secondary, higher education, graduate studies), maternal cohabitation (yes, no), maternal residence (capital area [Copenhagen], big cities [Aarhus, Aalborg, Odense], other), maternal income (no income, lowest tertile, middle tertile, highest tertile).

### Statistical analysis

Hazard ratios (HRs) with 95% confidence intervals (CIs) were estimated in Stata 11 using Cox proportional hazards regression with robust estimation, to account for some mothers in the cohort having more than one child. The child’s age was used as the underlying time axis. The assumption of proportional hazards was evaluated graphically.

### Ethical approval

The study was approved by the Danish Data Protection Agency (j nr. 2008-41-2680) and Scientific Ethics Committee of Central Jutland Region (VEK, sagnr. M-20100252).

## Results

The comprised of 2,123,228 children and 2.6% of the children (n = 56,016) were considered exposed (their mother received a diagnosis of cancer from between two years prior to pregnancy up to 50 years of age). There were 95,815 children (4.5%) who received any congenital anomaly diagnosis during their first year of life. Extending follow-up to cover the entire follow-up period (therefore including some more minor anomalies) resulted in 204,378 children (9.6%) with congenital anomalies. Of the 95,815 children with a congenital anomaly registered before 1 year of age, 9,408 (9.8%) had a sibling who also had a congenital anomaly registered prior 1 year of age; and 9,568 (10.0%) had more than one anomaly registered in their first year.

Table 1 displays the characteristics of the study population. Fewer exposed children were seen in the lowest tertile for maternal income; however there were more missing data among the exposed, especially for socioeconomic variables.

**Table 1:** Descriptive statistics of the study population by exposure status.

	Total (N = 2,123,228)	Exposed (N = 56,016)	Unexposed (N = 2,067,212)
Variables	N (%)	N (%)	N (%)
**Maternal age**			
<27	715,605 (34)	18,719 (33)	969,886 (34)
27–<30	647,976 (31)	16,921 (30)	631,055 (31)
≥31	759,474 (36)	20,373 (36)	739,101 (36)
Missing	173 (<1)	3 (<1)	170 (<1)
**Paternal age**			
<27	371,260 (17)	10,079 (18)	361,181 (17)
27–<30	560,459 (26)	14,816 (26)	545,643 (26)
≥31	1,122,201 (53)	29,750 (53)	1,092,451 (53)
Missing	69,308 (3)	1,371 (2)	67,937 (3)
**Maternal years of education**			
Lower secondary	494,442 (23)	13,445 (24)	480,997 (23)
Higher secondary	719,380 (34)	17,383 (31)	701,997 (34)
Higher education	390,655 (18)	9,714 (17)	380,941 (18)
Graduate studies	92,966 (4)	2,156 (4)	90,810 (4)
Missing	425,785 (20)	13,318 (24)	412,467 (20)
**Cohabitation**			
Yes	899,149 (42)	22,589 (40)	876,560 (42)
No	842,610 (40)	20,941 (37)	821,669 (40)
Missing	381,469 (18)	12,486 (22)	368,983 (18)
**Maternal residence**			
Capital area (Copenhagen)	468,086 (22)	11,506 (21)	456,580 (22)
Big cities (Aarhus, Aalborg, Odense)	221,849 (11)	5,607 (10)	216,242 (10)
Other	1,051,824 (50)	26,417 (47)	1,025,407 (50)
Missing	381,469 (18)	12,486 (22)	368,983 (18)
**Income**			
No income	50,599 (2)	1,143 (2)	49,456 (2)
Lowest tertile	374,214 (18)	7,467 (11)	366,747 (18)
Middle tertile	950,074 (45)	24,761 (44)	925,313 (45)
Highest tertile	366,855 (17)	10,159 (18)	356,696 (17)
Missing	381,486 (18)	12,486 (22)	369,000 (18)
**Parity**			
1	989,286 (47)	26,165 (47)	963,121 (47)
2	788,451 (37)	21,623 (39)	766,828 (37)
≥3	339,454 (16)	8,080 (14)	331,374 (16)
Missing	6,037 (<1)	148 (<1)	5,889 (<1)
**Birth weight**			
<2500g	96,613 (5)	2,931 (5)	93,682 (5)
2500–<3250g	499,294 (24)	13,267 (24)	486,027 (24)
3250–<4000g	939,531 (44)	23,944 (43)	915,587 (44)
≥4000g	314,523 (15)	7,808 (14)	306,715 (15)
Missing	273,267 (13)	8,066 (14)	265,201 (13)
**Gestational age**			
<37 weeks	150,099 (7)	4,158 (7)	145,941 (7)
≥37 weeks	1,973,129 (93)	51,858 (93)	1,921,271 (93)
**Apgar score at 5 minutes**			
0–7	29,730 (1)	789 (1)	28,941 (1)
8–9	106,942 (5)	2,697 (5)	104,245 (5)
10	1,764,734 (83)	46,715 (83)	1,718,019 (83)
Missing	221,822 (10)	5,815 (10)	216,007 (10)
**Singleton**			
Yes	1,923,209 (91)	51,864 (93)	1,871,345 (91)
No	65,521 (3)	1,614 (3)	63,907 (3)
Missing	134,498 (6)	2,538 (5)	131,960 (6)
**Sex**			
Male	1,089,210 (51)	28,700 (51)	1,060,510 (51)
Female	1,034,001 (49)	27,315 (49)	1,006,686 (49)
Missing	17 (<1)	1 (<1)	16 (<1)

The associations between maternal cancer and congenital anomalies in the offspring, diagnosed before 1 year of age, are shown in Table 2. When we compared the exposed group to the unexposed group, we observed an adjusted hazard ratio (HR) of 1.04 (95% confidence interval [CI]: 1.00, 1.09). The additional adjustment for socioeconomic variables resulted in a lower HR of 1.02 (95% CI: 0.98, 1.07). When considering timing of cancer diagnosis in relation to the in utero period, we observed a higher risk of congenital anomalies in children exposed to cancers diagnosed before or during pregnancy (HR: 1.37, 95% CI: 1.07, 1.75). Splitting this further to look at the period 2 years pre-pregnancy and the pregnancy period separately, we found HRs of 1.41 (95% CI 1.03, 1.93) and 1.33 respectively (95% CI 0.91, 1.94) (data not displayed). When considering exposure during the postpartum period, a statistically significant HR was seen only for children who were exposed to cancers diagnosed more than 10 years postpartum (HR: 1.06, 95% CI: 1.01, 1.16).

**Table 2:** Hazard ratios (HR) for all congenital anomalies diagnosed before 1 year of age according to exposure status.

Exposure status	Entire follow up period			
	Congenital anomaly cases (rate per 10,000 live births)	Crude HR	Model 1 adjusted HR (95% CI) ^a^	Model 2 adjusted HR (95% CI) ^b^
Not exposed	93,211 (451)	1 (ref)	1 (ref)	1 (ref)
Exposed	2,604 (465)	1.03	1.04 (1.00, 1.09)	1.02 (0.98, 1.07)
*Timing of exposure*				
Cancer diagnosed 2 years pre-pregnancy or during pregnancy	65 (620)	1.39	1.37 (1.07, 1.75)	1.33 (1.03, 1.71)
Cancer diagnosed up to 5 years postpartum	429 (490)	1.09	1.06 (0.97, 1.17)	1.04 (0.94, 1.15)
Cancer diagnosed after 5 years postpartum up to 10 years postpartum	534 (437)	0.97	0.97 (0.89, 1.06)	0.93 (0.85, 1.03)
Cancer diagnosed after 10 years postpartum	1,555 (465)	1.03	1.06 (1.01, 1.16)	1.05 (0.99, 1.11)
Cancer diagnosed in multiple time periods	21 (385)	0.85	0.89 (0.58, 1.35)	0.78 (0.48, 1.27)

^a^ Adjusted for maternal age group, paternal age group, birth year and sex

^b^ Additionally adjusted for socioeconomic variables at time of birth: maternal highest education, maternal cohabitation, maternal cohabitation, maternal residence, maternal income

Including all congenital anomalies diagnosed throughout the entire follow-up period resulted in 204,378 children with diagnoses (9.6% of the study population) and gave a HR of 1.03 (95% CI: 0.99, 1.05) (data not displayed). The sensitivity analysis using 45 years of age as the maximum cut off for maternal diagnosis of cancer resulted in a similar HR of 1.05 (95% CI: 1.00, 1.10) (data not displayed).

Using paternal cancer diagnosed during the same window as the exposure gave HR results close to that of the main analysis (HR: 1.08, 95% CI: 1.02, 1.14). We further assessed timing of cancer diagnosis in relation to the *in utero* period and we observed a similar pattern for HRs with regards to timing: pre-pregnancy or during pregnancy HR 1.26 (95% CI: 1.02, 1.55); in the 5 years postpartum HR 0.97 (95% CI: 0.86, 1.09); >5 years to 10 years postpartum HR 1.14 (95% CI: 1.04, 1.26); >10 years postpartum HR 1.07 (95% CI: 0.99, 1.16); or in multiple time points HR 1.07 (95% CI: 0.61, 1.87).

In Table 3 results are shown for specific groups of congenital anomalies among the exposed during the entire follow-up period compared with the unexposed. The largest point estimates were seen for congenital anomalies of the musculoskeletal system (HR: 1.13, 95% CI: 1.02, 1.25) and congenital anomalies of the respiratory system (HR: 1.11, 95% CI: 0.88, 1.41),. Among the exposed, the most common congenital anomalies in these groups were congenital anomalies of the feet (39%) and of the larynx, trachea and bronchus (67%) respectively. The lowest point estimates were seen for congenital anomalies of the nervous system (HR: 0.80, 95% CI: 0.61, 1.04), orofacial clefts (HR: 0.82, 95% CI: 0.55, 1.23) and congenital anomalies of the genital organs (HR: 0.87, 95% CI: 0.68, 1.1).

**Table 3:** Hazard ratios (HR) for congenital anomaly groupings according to exposure.

Congenital anomalies		Entire follow up period			
		Cases	Crude HR	Model 1 Adjusted HR (95% CI) ^a^	Model 2 Adjusted HR (95% CI) ^a^^,^^b^
Nervous system	Unexposed	3,922	1.0 (ref)	1.0 (ref)	1.0 (ref)
	Exposed	123	0.88	0.80 (0.61, 1.04)	0.74 (0.53, 1.03)
Eye, ear or nose	Unexposed	4,070	1.0 (ref)	1.0 (ref)	1.0 (ref)
	Exposed	95	0.90	0.96 (0.77, 1.20)	0.87 (0.66, 1.14)
Circulatory system	Unexposed	19,852	1.0 (ref)	1.0 (ref)	1.0 (ref)
	Exposed	567	0.86	1.00 (0.76, 1.33)	0.98 (0.71, 1.34)
Respiratory system	Unexposed	3,676	1.0 (ref)	1.0 (ref)	1.0 (ref)
	Exposed	92	0.86	1.11 (0.88, 1.41)	1.14 (0.87, 1.48)
Orofacial clefts	Unexposed	3,859	1.0 (ref)	1.0 (ref)	1.0 (ref)
	Exposed	100	0.75	0.82 (0.55, 1.23)	0.79 (0.49, 1.25)
Digestive system	Unexposed	10,104	1.0 (ref)	1.0 (ref)	1.0 (ref)
	Exposed	289	1.10	1.03 (0.90, 1.19)	1.00 (0.85, 1.17)
Genital organs	Unexposed	10,478	1.0 (ref)	1.0 (ref)	1.0 (ref)
	Exposed	264	0.67	0.87 (0.68, 1.10)	0.85 (0.66, 1.10)
Urinary system	Unexposed	4,114	1.0 (ref)	1.0 (ref)	1.0 (ref)
	Exposed	95	0.74	0.93 (0.71, 1.23)	0.97 (0.73, 1.29)
Musculoskeletal system	Unexposed	36,409	1.0 (ref)	1.0 (ref)	1.0 (ref)
	Exposed	1,049	0.84	1.13 (1.02, 1.25)	1.14 (1.02, 1.27)
Other congenital anomalies and chromosomal abnormalities	Unexposed	9,096	1.0 (ref)	1.0 (ref)	1.0 (ref)
	Exposed	250	0.89	1.02 (0.81, 1.27)	0.99 (0.78, 1.27)

^a^ Adjusted for maternal age group, paternal age group, birth year and sex

^b^ Additionally adjusted for socioeconomic variables at time of birth: maternal highest education, maternal cohabitation, maternal cohabitation, maternal residence, maternal income

Table 4 displays results for all congenital malformations, according to the type of cancer the mother was diagnosed with. Statistical significance was reached for children whose mothers were diagnosed with leukaemia (HR: 1.31, 95% CI: 1.01, 1.69). The largest, insignificant point estimates were seen for cancer of the lip, oral cavity or pharynx (HR: 1.15, 95% CI: 0.83, 1.59) and cancer of the respiratory or intrathoracic organs (HR: 1.18, 95% CI: 0.99, 1.39). Among cases, the most common type of lip, oral or pharynx cancer they were exposed to was cancer of the tonsil (24%) and the most common type of respiratory or intrathoracic cancer they were exposed to was cancer of the bronchus and lung (88%). Largest, but statistically insignificant negative HRs were seen for cancer or the bone or articular cartilage (HR: 0.71, 95% CI 0.29, 1.76) and cancer of the endocrine glands (HR: 0.83, 95% CI: 0.66, 1.04).

**Table 4:** Hazard ratios (HR) for any congenital anomaly diagnosis before 1 year of age according to type of cancer.

Type of cancer		Entire follow up period			
		Cases	Crude HR	Model 1: Adjusted HR (95% CI) ^a^	Model 2:Adjusted HR (95% CI) ^a^^,^^b^
Lip, oral cavity or pharyngeal	Unexposed	95,773	1.0 (ref)	1.0 (ref)	1.0 (ref)
	Exposed	42	1.13	1.15 (0.83, 1.59)	1.15 (0.80, 1.65)
Digestive organs	Unexposed	95,626	1.0 (ref)	1.0 (ref)	1.0 (ref)
	Exposed	189	0.99	1.01 (0.87, 1.16)	1.00 (0.85, 1.18)
Respiratory or intrathoracic organs	Unexposed	95,679	1.0 (ref)	1.0 (ref)	1.0 (ref)
	Exposed	136	1.13	1.18 (0.99, 1.39)	1.11 (0.91, 1.37)
Bone or articular cartilage	Unexposed	95,809	1.0 (ref)	1.0 (ref)	1.0 (ref)
	Exposed	6	0.69	0.71 (0.29, 1.76)	0.82 (0.34, 2.04)
Skin	Unexposed	95,470	1.0 (ref)	1.0 (ref)	1.0 (ref)
	Exposed	345	0.99	0.99 (0.89, 1.11)	0.97 (0.86, 1.08)
Mesothelial or soft tissue	Unexposed	95,778	1.0 (ref)	1.0 (ref)	1.0 (ref)
	Exposed	37	1.03	1.03 (0.74, 1.44)	1.02 (0.72, 1.44)
Breast	Unexposed	94,798	1.0 (ref)	1.0 (ref)	1.0 (ref)
	Exposed	1,017	1.02	1.04 (0.98, 1.11)	1.02 (0.95, 1.09)
Female genital organs	Unexposed	95,318	1.0 (ref)	1.0 (ref)	1.0 (ref)
	Exposed	497	1.06	1.06 (0.97, 1.17)	1.03 (0.93, 1.15)
Urinary tract	Unexposed	95,788	1.0 (ref)	1.0 (ref)	1.0 (ref)
	Exposed	27	0.89	0.92 (0.63, 1.35)	0.90 (0.59, 1.36)
Central nervous system	Unexposed	95,799	1.0 (ref)	1.0 (ref)	1.0 (ref)
	Exposed	16	1.05	1.07 (0.87, 1.32)	1.04 (0.81, 1.34)
Endocrine glands	Unexposed	95,745	1.0 (ref)	1.0 (ref)	1.0 (ref)
	Exposed	70	0.83	0.83 (0.66, 1.04)	0.86 (0.67, 1.09)
Lymphoid or haematopoietic	Unexposed	95,804	1.0 (ref)	1.0 (ref)	1.0 (ref)
	Exposed	11	1.06	1.11 (0.64, 1.92)	1.22 (0.65, 2.28)
Leukaemia	Unexposed	95,754	1.0 (ref)	1.0 (ref)	1.0 (ref)
	Exposed	61	1.33	1.31 (1.01, 1.69)	1.28 (0.96, 1.70)

^a^ Adjusted for maternal age group, paternal age group, birth year and sex

^b^ Additionally adjusted for socioeconomic variables at time of birth: maternal highest education, maternal cohabitation, maternal cohabitation, maternal residence, maternal income

## Discussion

Maternal cancer diagnosis in the two years before pregnancy and the years after pregnancy was associated with a slightly higher risk of congenital anomalies in children. The highest risk was seen children of mothers who were diagnosed before or during pregnancy, with smaller risk estimates in those whose mothers were diagnosed within 5 years postpartum or after 10 years postpartum. Similar associations were seen for paternal cancers which indicate that maternal cancer does not influence risk of offspring congenital anomalies, and genetic factors or other time stable factors, like socio-economic factors, play a role instead.

Immortal observation time bias and reverse causation should also be considered. To be diagnosed up to the age of 50 years, the mother needs to stay alive. Some of the cancers may have been diagnosed following increased monitoring because of the presence of a congenital anomaly in the offspring (although it could be argued that this is unlikely). The first type of bias may lead to an underestimation of effects, and the second type to an overestimation; but both types of bias would only have small effects.

While the majority of previous studies have not reported an association [7;13;14], some studies have suggested an increase in prevalence of congenital anomalies among offspring of mothers diagnosed with pregnancy-associated cancers [7;11;12]. Similar to our study, positive associations have been seen for all cancers [11],breast cancers [5] and lymphomas [12]. The comparisons that can be drawn between our results and those of previous studies are, however, limited due to limited power and differences in the exposure definitions: we were unable to distinguish between major and minor congenital anomalies, and other studies have limited the exposure window to pregnancy or from conception up to two years postpartum.

The associations seen with cancers diagnosed during pregnancy and the pre-pregnancy period (the time period in which we observed the highest HR) could be due to cancer treatments or stress following a diagnosis, rather than the cancer itself. Cancers diagnosed soon after pregnancy would have been more likely to be initiated before or during gestation than cancers initiated many years after; and, therefore, larger HRs would have been expected in the earlier postpartum years if the cancer itself and its metabolic consequences had an effect on offspring congenital anomaly risk. There was not, however, a clear pattern with regards to risk and timing of maternal cancer diagnosis.

The similar pattern of results seen when paternal cancer was substituted as the exposure suggests that any increase in congenital anomalies in the offspring is not specific to cancers in the mother; rather, it could be an indication that uncontrolled genetic or family-related confounding factors are causing the observed association. If a true association exists between maternal cancer and also paternal cancer, and congenital anomalies in the children, this lends support to the theory of shared genetic and pre-pregnancy environmental factors, as suggested by Zhu et al [15]. An alternative mechanism we discussed was the presence and development of maternal cancer resulting in nutritional and metabolic challenges which fetal morphogenesis or organogenesis; in this scenario a positive association would be unexpected in the analysis on paternal cancer.

The main strength of this study is the use of complete data from the Danish nationwide register system. The registers cover the whole of Denmark. This provides data on the entire population, with minimal selection bias. In the study of specific types of congenital anomalies or types of maternal cancers, numbers are small, resulting in a lack of power, which can be seen from the wide confidence intervals for some of our analyses. Cancer data are considered to be of high quality and completeness [28]. Data on congenital anomalies in the Danish National Patient Register is considered to be 90% complete; a level considered by some to be acceptable for some types of research [30]. Completeness would be expected to be poorer in the earlier years of the data and for minor congenital anomalies, especially if they are not visible. We were unable to distinguish between major and minor congenital anomalies in our data, however restricting the main analyses to congenital anomalies diagnosed in the first year of life will most likely limit these analyses to more major anomalies.

Although the inclusion of the entire population of Denmark minimised selection bias, the study only includes information on live births. As major congenital anomalies are associated with early fetal loss, stillbirth and termination [31–33], it is possible that bias occurs due to conditioning on live births [34]. Bias due to differences in terminations between the exposed and unexposed groups would be more likely to be an issue when considering children of mothers who received a diagnosis during pregnancy or the pre-pregnancy period, who may be more likely to terminate a pregnancy due to concerns about a fetotoxic treatment. It is less likely that there would be a link between postpartum diagnoses and termination. Bias due to early fetal loss and stillbirth could occur when considering any exposure category, if losses or prenatal screening for congenital anomalies are more common among mothers with cancer at any stage of development.

Another limitation of the study is the fact that it conditions on the future. Although maternal cancer susceptibility will exist and a tumour may already be initiated before a child's birth, the exposure status of the child is based on a future event: diagnosis of a maternal cancer. It is unlikely that cancer would be differentially diagnosed among mothers who have a child with a congenital malformation. However, in order for a diagnosis to be made and a child considered exposed, mothers have to survive long enough to receive a cancer diagnosis, not dying of alternative causes before this point.

There is potential for misclassification of exposure status due to incomplete exposure follow-up. Children were considered exposed if their mother was diagnosed with cancer from two years before conception, up until the mother reached 50 years of age. However, not all mothers in the cohort reached 50 years before December 31st 2010, the final date cancer data were available until, and some children will therefore have been incorrectly classified as unexposed in some analyses. However, the sensitivity analysis, reducing the maximum maternal age to 45 years, provided similar results.

There was some suggestion of an increased prevalence of congenital anomalies among children of mothers who were diagnosed with cancer between 2 years prior to pregnancy and in the years after pregnancy (up to 50 years of age). However, the finding of a positive association between paternal cancer and congenital anomalies suggest that genetic factors or environmental factors, in either the mother or the father, prior to conception influence risk. The presence of a developing maternal cancer whilst the offspring is *in utero* may not greatly alter congenital anomaly risk, at least for those who survive until birth. Our results should be interpreted in light of the limitations of the study. Additionally, it should be considered that a large number of analyses were carried out and some chance findings cannot be ruled out. With a larger sample size, it may be interesting to investigate associations between more specific types of cancer and specific congenital malformations to further consider some of our results.
